# Association between Dietary Pattern, Weight Loss, and Diabetes among Adults with a History of Bariatric Surgery: Results from the Qatar Biobank Study

**DOI:** 10.3390/nu16142194

**Published:** 2024-07-10

**Authors:** Ruba Almaghrbi, Razan Alyamani, Lama Aliwi, Joyce Moawad, Akhtar Hussain, Youfa Wang, Zumin Shi

**Affiliations:** 1 Department of Human Nutrition, College of Health Sciences, Qatar University, Doha P.O. Box 2713, Qatar; ra1905998@student.qu.edu.qa (R.A.); ra1904667@student.qu.edu.qa (R.A.); la1808270@student.qu.edu.qa (L.A.); jmoawad@qu.edu.qa (J.M.); 2 Faculty of Health Sciences, Nord University, 8049 Bodø, Norway; 3 International Diabetes Federation, 166 Chaussee de La Hulpe, B-1170 Brussels, Belgium; 4 Xi’an Jiaotong University Health Science Center, Global Health Institute, Xi’an 710061, China; youfawang@xjtu.edu.cn

**Keywords:** bariatric surgery, diabetes mellitus, dietary patterns, glycemic control, Qatar Biobank study, adults

## Abstract

We aimed to examine the association between weight loss, dietary patterns, diabetes, and glycemic control among Qatari adults with a history of bariatric surgery (BS). Data from 1893 adults from the Qatar Biobank study were analyzed. Diabetes was defined by blood glucose, HbA1c, and medical history, with poor glycemic control defined as HbA1c ≥ 7.0%. The dietary patterns were derived from a Food Frequency Questionnaire using factor analysis. The participants’ mean age was 38.8 years, with a mean weight loss of 23.4% and a 6.1% prevalence of poor glycemic control. Weight loss was inversely associated with diabetes and poor glycemic control. The traditional dietary pattern (high intake of Biryani, chicken, meat, fish dishes, zaatar fatayer, croissant, lasagna, and Arabic bread) was inversely associated with diabetes prevalence, with an OR of 0.61 (95%CI, 0.41–0.99) when comparing extreme quartiles. No significant associations were found between prudent or sweet dietary patterns and diabetes. Among the individuals with known diabetes, the prevalence of remission was 33.4%, with an OR for remission of 5.94 (95%CI, 1.89–18.69) for the extreme quartiles of weight loss. In conclusion, weight loss and traditional dietary patterns are inversely associated with diabetes and glycemic control among adults with a history of BS, with weight loss being the main determinant.

## 1. Introduction

Obesity, characterized by excessive accumulation of body fat, is a global public health crisis. According to the 2024 World Obesity Atlas Report, 42% of the world adult population was overweight or obese in 2020 [1]. Linked with various non-communicable diseases (NCDs), such as type 2 diabetes (T2DM), hypertension, cardiovascular diseases, asthma, Alzheimer, metabolic syndrome, liver steatosis, as well as increased mortality [2], obesity requires a comprehensive approach for its management, including diet adjustment, physical activity, behavioral intervention, pharmacotherapy, and bariatric surgery (BS) [3]. 

The alarming rise in the prevalence of obesity contributes to a vicious cycle with T2DM [4]. According to the International Diabetes Federation, the number of adults diagnosed with T2DM reached 537 million in 2021, with projections suggesting a surge to 643 million cases by 2030. This trend is particularly concerning in the Middle East region, where the prevalence of the disease is notably high [5]. Recent findings from a Qatar Biobank study (QBB) revealed a T2DM prevalence of 17.4% in the State of Qatar [6]. Moreover, based on a 2021 Global Burden of Disease Study, Qatar stands out with the highest age-specific diabetes prevalence globally, notably recording 76.1% (73.1–79.5) among individuals aged 75–79 years [7]. However, more than half of individuals with diabetes had poor glycemic control in the Middle East region [8,9,10]. 

An effective approach for treating obesity is BS, which has been associated with remarkable benefits, such as remission of T2DM and weight loss [11,12,13]. The number of BSs conducted worldwide is increasing annually and has exceeded 685,000 [14]. In Qatar, recent data from the Qatar Biobank (QBB) study indicated that the prevalence of BS was 12% in the general population [6]. 

BS leads to a reduction in energy intake and a decrease in food tolerance that are attributed to altered appetite regulation, a significant reduction in stomach size, and the occurrence of gastrointestinal symptoms, such as the dumping syndrome [12,15,16,17]. There is also a change in food preference after BS [17,18,19,20]. In clinical trials, BS has been linked to a 30% weight loss from the baseline a decade after the procedure and can achieve short-term diabetes remission [21]. A high proportion of BS patients do not follow the dietary guidelines, which may lead to unsatisfactory weight reduction and corresponding metabolic problems among BS patients [19,22,23]. 

A meta-analysis demonstrated that adopting healthy eating habits can improve insulin sensitivity, thereby reducing diabetes-related complications [24]. In a cross-sectional study conducted in Qatar, an inverse association was observed between the modern dietary pattern and poor glycemic control, especially in men [10]. 

Dietary habits play a crucial role in the development of obesity and T2DM, both recognized as primary determinants of cardiovascular disease (CVD) [25]. A meta-analysis conducted in 2023, showed that ketogenic, Mediterranean, moderate-carbohydrate, and low-glycemic-index diets are beneficial for managing diabetes, highlighting the influence of weight loss as a mediator of the association between diet and glycemic control among individuals with diabetes [26].

There is a lack of data on the dietary patterns of individuals after BS and their association with glycemic control in Qatar and in the Gulf states. Thus, this study aimed to identify dietary patterns after BS. We also examined the association between dietary patterns and diabetes, as well as glycemic control among adults in Qatar. Furthermore, the association between weight loss after BS and diabetes was assessed.

## 2. Materials and Methods

### 2.1. Study Sample

The QBB study, started in 2012, is an ongoing cohort study conducted among the general population. It enrolls individuals who are Qatari or long-term residents (≥15 years). The recruitment of participants is conducted through various channels, such as the internet, personal networks (family and friends), or social media platforms. By 2022, more than 20,000 adults had enrolled in the QBB study. This study comprises a total of 1893 (713 males and 1180 females) QBB participants who were aged 18 years old and above, had a history of BS, information on dietary intake, and diabetes-related measures. Ethical approval for the QBB study was obtained from the Ethics Committee of Hamad Medical Corporation in 2011 and continued with the QBB Institutional Review Board (IRB) from 2017. All participants gave written informed consent before participation. The current study was approved under the IRB exempted category (Ex-2023-QF-QBB-RES-ACC-00136-0235).

### 2.2. Outcome Variables: Diabetes and Poor Glycemic Control

Diabetes was defined as fasting blood glucose ≥ 7 mmol/L or random blood glucose ≥ 11.0 mmol/L, or HbA1c ≥ 6.5%, or self-reported diabetes [27]. Poor glycemic control was defined as having an HbA1C level of 7.0% or higher.

Diabetes remission was defined as HbA1c < 6.5% and a negative answer to the question “Are you being treated for your diabetes with insulin or tablets”. 

### 2.3. Independent Variables: Dietary Patterns and Weight Loss

To assess dietary intake, participants completed a self-administered computerized Food Frequency Questionnaire (FFQ). The FFQ collects participants’ dietary habits, frequency of food and beverage consumption, and any modifications they have made to their diet over the past year. The FFQ included 102 food items adapted from the European Perspective Investigation into Cancer and Nutrition (EPIC) study, but it has not been validated for use in Qatar. The food items were grouped into 38 food groups based on similarities in nutrient profiles and cooking methods. The frequency of intake (times per week) for each of the 38 food groups was used as input for factor analysis to construct dietary patterns. The criteria used to determine the number of dietary patterns included an eigenvalue greater than one, a scree plot, and patterns that were interpretable based on the food culture in Qatar. Varimax rotation was used to help interpret the identified patterns. Pattern-specific factor scores were assigned to each participant. The scores were calculated by summing the products of the factor loading coefficients and the standardized weekly frequency intake of each food group associated with that pattern.

Weight loss was calculated based on current weight and the weight before BS. Both the absolute weight loss and the percentage of weight loss were recoded as quartiles in the analyses.

### 2.4. Covariates

We consider the following covariates in this study: age, gender, leisure time physical activity level (the metabolic equivalent of task (MET), recoded as tertiles), smoking (self-reported and recoded as non-smokers, ex-smokers, and current smokers), sleep duration, and snoring. The weight and height of the participants were measured using a digital scale and a Seca stadiometer, respectively, by the research nurses. The body mass index (BMI) was then computed as the weight in kilograms divided by the square of height in meters (kg/m^2^). Medication use was self-reported. Depression syndrome was based on a Patient Health Questionnaire (PHQ-9) score of ≥10. 

The time when bariatric surgery was conducted was not asked in this study. However, the participants were asked about where the bariatric surgery was conducted and was further categorized as in Qatar or outside Qatar. The location of bariatric surgery was used as an indicator of how long before the BS was conducted. In general, the BSs conducted outside Qatar were earlier than those conducted in Qatar, as most of the BS surgeries were conducted after 2011 [28].

### 2.5. Statistical Analysis

The continuous variables were presented as means and standard deviations, while the categorical variables were described using frequencies and percentages. The χ^2^ test was used to compare differences between groups for categorical variables and an ANOVA or Kruskal–Wallis test was used for the continuous variables, based on the distribution of the data. Multivariable logistic regression models were used to examine the association between dietary patterns and diabetes or poor glycemic control. A set of three models was used: model 1 adjusted for age and gender; model 2 further adjusted for smoking, education, physical activity, sleep duration, and snoring; and model 3 further adjusted for quartiles of weight loss. 

Subgroup analyses were conducted, and the multiplicative interactions were tested. The marginsplot command was used to visualize the interaction between dietary patterns and insulin use in relation to glycemic control. The association between weight loss and diabetes, poor glycemic control, or diabetes remission was assessed using logistic regression with the adjustment for age, gender, education, smoking, physical activity, hypertension, sleep duration, snoring, and dietary patterns. We did not perform multiple imputation for the missing values due to the small number of missing values. Instead, we provided the number of missing values in the tables.

All the analyses were performed using STATA 18 (Stata Corporation, College Station, TX, USA). Statistical significance was considered when *p* < 0.05 (two-sided).

## 3. Results

### 3.1. Sample Description

The mean age of the participants was 38.8 (SD 10.6) years. Overall, the prevalence of T2DM among all participants was 19.4%, while the prevalence of poor glycemic control was 6.1%, and 21.7% of participants were insulin users (23.1% of men and 21.7% of women). The prevalence of undiagnosed diabetes was low (19.4% − 18.5% = 0.9%). In total, 75.3% of participants were on a diet, with an obesity prevalence of 54.9%. The mean percentage of weight loss was 23.4% (SD 13.1). Figure S1 illustrates the distribution of weight loss among the participants. Among the participants, 42.3% reported that their bariatric surgery was conducted outside Qatar (53.5% of men and 35.5% of women). More than 60% of participants had a sleep duration of less than 7 h. The overall leisure time physical activity was low, with a median of 6.0 (IQR 0–25.5) MET hours/week.

### 3.2. Dietary Patterns

Three dietary patterns were identified (Table S1). Factor 1, identified as traditional, consisted of biryani (البرياني), chicken, meat, and fish dishes, zaatar fatayer (فطائر بالزعتر), croissant, lasagna, and Arabic bread (خبز عربي). Factor 2, named prudent, was characterized by high loadings of fresh fruits, salad, raw vegetables, canned and dried fruits, dates (تمر), grilled, fried, or baked fish, and fresh juice. Factor 3, the “Sweet and fast food” pattern, included desserts, ice cream, chocolate, fast food, and nuts. These three factors accounted for 24.2%, 5.9%, and 4.9% of the variance in food intake, respectively. 

Table 1 presents the sample characteristics by quartiles of traditional pattern intake. A higher intake of the traditional dietary pattern was associated with a younger age compared to those with a lower intake (35.8 vs. 40.8 years). In comparison to a lower intake of the traditional pattern, a higher intake was associated with lower education, a normal BMI, good glycemic control, and a greater percentage of weight loss.

### 3.3. Association between Different Dietary Patterns and Glycemic Control Post BS

The traditional dietary pattern was inversely associated with diabetes (Table 2). After adjusting for sociodemographic and lifestyle factors, compared with quartile one, quartile four of the traditional dietary pattern had an odds ratio (95%CI) for diabetes of 0.61 (0.41–0.99). The association remained after further adjusting for weight loss. No significant association was found between the prudent diet and the sweet diet with diabetes. 

The traditional diet was also inversely associated with poor glycemic control; comparing the extreme quartiles of the traditional dietary pattern, the OR for poor glycemic control was 0.52 (95% CI 0.27–1.00) after adjusting for sociodemographic and lifestyle factors (Table 3). However, this association was attenuated after further adjusting for weight loss.

### 3.4. Subgroup Analysis

No significant interaction was found between the traditional dietary pattern and sociodemographic and lifestyle factors. However, the inverse association between the traditional dietary pattern and poor glycemic control was mainly observed in those with older age, high education, or in those who had bariatric surgery conducted outside Qatar (Table 4). A strong inverse linear association between the traditional dietary patter and poor glycemic control was found among those had bariatric surgery conducted outside Qatar. 

The prudent pattern was positively associated with poor glycemic control among people who were less than 40 years old (Q4 vs. Q1: OR 3.89 (1.15–13.17)), while in older individuals, there was no significant association. There were no significant associations between the sweet/fast pattern and poor glycemic control. 

**Figure 1:**
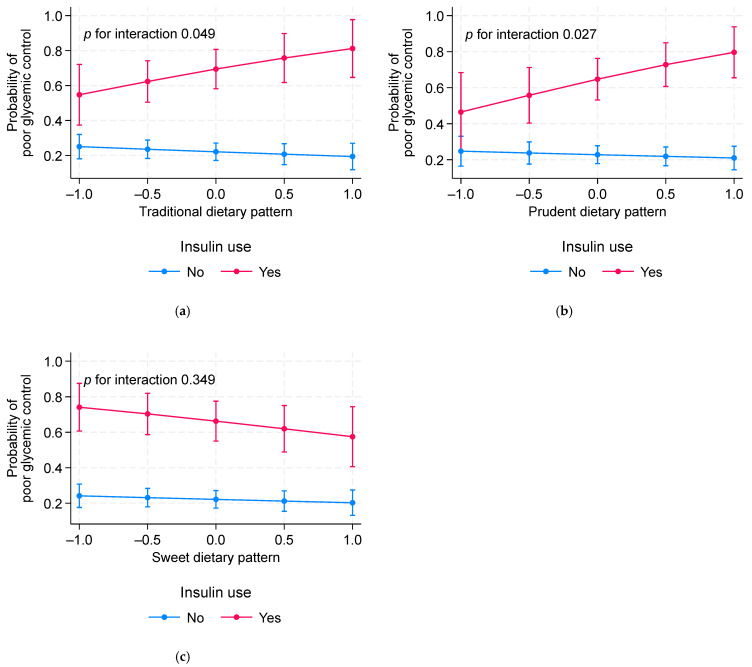
Interaction between dietary pattern and insulin use in relation to poor glycemic control among adults with known diabetes. (**a**) traditional dietary pattern (**b**) prudent dietary pattern (**c**) sweet dietary pattern. Values are probability of poor glycemic control (95%CI) calculated using the margins command in Stata after a logistic regression model. Models are adjusted for age, sex, education, smoking, physical activity, hypertension, sleep duration, and snoring.

Among those with known diabetes, there was a significant interaction between the prudent pattern, the traditional pattern, and insulin use in relation to poor glycemic control (Figure 1). Both the prudent dietary pattern and the traditional pattern were positively associated with poor glycemic control among the insulin users.

### 3.5. Association between Weight Loss and Diabetes and Poor Glycemic Control

**Figure 2:**
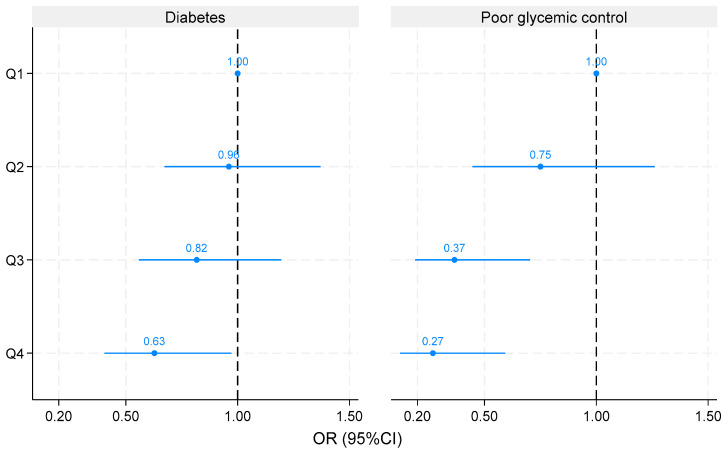
Association between quartiles of weight loss and diabetes and poor glycemic control among adults with a history of bariatric surgery who attended the Qatar Biobank study. Models are adjusted for age, sex, education, smoking, physical activity, hypertension, sleep duration, snoring, and dietary patterns. Values are odds ratios (95%CI). Mean weight loss was 7.3, 20.8, 31.2, and 51.3 kg across the quartiles of weight loss.

Weight loss was inversely associated with diabetes and poor glycemic control (Figure 2). Across the quartiles of weight loss, the odds ratios (95%CI) for diabetes were 1.00, 0.96 (0.67–1.37), 0.82 (0.56–1.20), and 0.63 (0.41–0.97), respectively. The corresponding figures for poor glycemic control were 1.00, 0.75 (0.45–1.26), 0.37 (0.19–0.70), and 0.27 (0.12–0.59), respectively. 

Among the 350 individuals with history of diabetes, the prevalence of diabetes remission was 33.4% (Table S2). Among all the factors examined, weight loss was the strongest predictor for diabetes remission (Table S3). Comparing the extreme quartiles of weight loss, the OR for diabetes remission was 5.94 (95%CI, 1.89–18.96). 

### 3.6. Diabetes Medication Use and Glycemic Control among Individuals with Known Diabetes

Table S4 shows that among the individuals with known diabetes (*n* = 311), moderate weight loss was a strong predictor for reduced likelihood of using insulin (95% CI, OR = 0.34 (0.15–0.81), *p* = 0.015), other diabetes medication use (OR = 0.47 (0.23–0.97), *p* = 0.040), and improved glycemic control (OR = 0.28 (0.13–0.61), *p* = 0.001). However, dietary patterns were not significantly associated with either medication use or glycemic control, except for individuals following a sweets/fast food diet, who had significantly lower odds of using other diabetes medications (OR = 0.63 (0.46–0.85), *p* = 0.003).

## 4. Discussion

In this large population study of patients with a history of bariatric surgery, we found that there was substantial weight loss among participants post BS (23.4%). A significant amount of weight loss was the main determinant of diabetes remission and glycemic control. The traditional dietary pattern was inversely associated with diabetes and glycemic control. Overall, no association between the prudent pattern and between the sweet/fast food pattern and diabetes was found. The participants following the sweet/fast food pattern had significantly lower odds of using other diabetes medications. 

### 4.1. Weight Loss Post-BS and Its Association with Diabetes

The percentage of weight loss among this study population (23.4%) was comparable to findings in other studies (12–56%) after BS [29,30]. The association between weight loss and diabetes and glycemic control in our study is in line with findings from other studies [31,32,33,34,35]. BS helps diabetes remission through significant weight loss, resulting from various mechanisms, such as caloric restriction, appetite regulation, altered taste, and altered gut–brain signaling pathways [36]. BS results in reduced hunger hormones (e.g., ghrelin) and increased satiety hormones, such as GLP-1 and PYY. These hormonal changes further inhibit glucagon and improve insulin secretion via the pancreatic β-cell GLP-1 receptors, thus improving glycemic control [12,15,17,37,38].

### 4.2. Dietary Pattern and Diabetes Post-BS

Comparison with other studies: the association between dietary intake after BS and glycemic control is inconclusive. A recent systematic review suggested that dietary patterns did not significantly influence weight loss post-surgery [39]. However, a narrative review that assessed the adherence to the Mediterranean diet pre- and post-BS found that this type of diet affects weight loss after the surgery and improves diabetes and glycemic control [40]. The original studies included in the two reviews had small sample sizes (mostly <100 patients in each study) [39,40]. The four studies included in the narrative review had sample sizes ranging from 37 to 78 [40]. Unlike existing studies, we found that the traditional dietary pattern was inversely associated with diabetes and poor glycemic control, especially among those reporting BS surgery outside Qatar. Although we do not have information on the years following BS, the information on the location where the BS was conducted can serve as a surrogate, as BS started in Qatar in 2011 [28]. Before 2011, a high number of Qatari residents went overseas for BS. 

The traditional dietary pattern in our study has both healthy (e.g., brown bread, tea, salad) and unhealthy food items (e.g., white rice, white bread, and fast food). The mechanisms of the observed inverse association need to be further examined. The traditional dietary pattern may be an indicator of a traditional lifestyle and a better social network. It could also be due to reverse causation: those with a better control of diabetes increased their intake of the traditional diet. Reverse causation could also be the explanation for the positive association between the prudent dietary pattern and poor glycemic control among insulin users in this study. To the best of our knowledge, no study has examined the association between dietary patterns constructed using factor analysis and glycemic control among those with a history of bariatric surgery. 

Postoperative effects on food consumption: BS has been shown to have postoperative effects on food consumption through reductions in energy intake, increased satiety, and decreased hedonic hunger and food tolerance [17]. However, it is debatable whether these changes led to long-term preference for or avoidance of food items [17]. The findings of an RCT indicated an increase in the consumption of protein-rich foods and a decrease in the intake of high-fat foods [17,20]. Sugar intake was shown to be reduced significantly after BS [20,22]. However, a recent systematic review stated that BS may lead to imbalanced dietary patterns, insufficient consumption of proteins, and excessive fat intake [41]. Moreover, patients tend to have insufficient dietary fiber intake (ranging from 10 g–22 g/d), in comparison to the recommended levels for healthy individuals [17]. Several studies found that the dietary intake of various micronutrients did not meet recommended levels such as vitamins D, E, C, folate, B12, B1, as well as essential minerals such as copper, zinc, calcium, and iron. Over time, the compliance with supplement intake showed a decline, with approximately 20–32% of patients reporting that they either ceased taking or never initiated their prescribed supplements within one year following the surgery [41]. In our study, the food composition of the three identified dietary patterns was similar to that in the general population in Qatar. Overall, based on factor loadings, avoiding unhealthy food items was not observed in this study. Further qualitative research is needed to understand the existence of the dietary patterns in the BS patients.

### 4.3. Weight Loss and Medication Use Post-BS

In this study, among participants with known diabetes, moderate weight loss had lower likelihood of insulin use. This result is consistent with current knowledge. High rates of postoperative cessation of insulin therapy among patients with T2DM were observed in large population studies [42,43]. 

### 4.4. Strengths and Limitations

Our study has several strengths. The incorporation of a large sample from the general population strengthens the validity of our results. The large sample size allowed us to conduct various subgroup analyses. Furthermore, blood samples were measured to assess the HbA1c levels. 

The main limitation of this study is that the duration following the BS was not available. Another limitation pertains to participants’ self-reporting of diabetes status, with insufficient information regarding the timeframe of diagnosis prior to or after the BS. Moreover, the use of FFQ without precise portion-size information is another limitation. Consequently, we could not assess the actual amounts of food consumed or adjust for energy intake in our analyses. In this study, the history of medication use was unknown. Diabetes remission was based on HbA1c and the status of current diabetes medication use instead of medication use within three months, which is commonly used to define diabetes remission. As the level of physical activity was low in the whole sample, it is difficult to test whether patients who engaged in physical activity had a faster beneficial response post-BS.

## 5. Conclusions

Bariatric surgery has shown positive outcomes in terms of weight loss and T2DM. Weight loss is the main determinant of diabetes and glycemic control. Similar dietary patterns were identified in adults with a history of bariatric surgery, as compared to the general population. The traditional dietary pattern was inversely associated with diabetes and glycemic control. Further research is needed to explore the long-term effects of post-BS dietary patterns on glycemic control. Promoting healthy eating habits should continue to be encouraged, especially for individuals taking diabetes medications. Maintaining a healthy weight should be the primary measure for diabetes prevention. 

## Figures and Tables

**Table 1:** Sample characteristics by quartiles of traditional dietary patterns among adults with a history of bariatric surgery who attended the Qatar Biobank study.

		Quartiles of Traditional Dietary Pattern				
	Total	Q1 (Low)	Q2	Q3	Q4 (High)	*p*-Value
N	1893 (100.0%)	474 (25.0%)	473 (25.0%)	473 (25.0%)	473 (25.0%)	
Age	38.8 (10.6)	40.8 (10.9)	40.4 (11.1)	38.1 (10.3)	35.8 (9.0)	<0.001
Gender						
Male	713 (37.7%)	153 (32.3%)	164 (34.7%)	209 (44.2%)	187 (39.5%)	<0.001
Female	1180 (62.3%)	321 (67.7%)	309 (65.3%)	264 (55.8%)	286 (60.5%)	
Education						
Low	300 (15.8%)	81 (17.1%)	78 (16.5%)	76 (16.1%)	65 (13.7%)	0.002
Medium	662 (35.0%)	145 (30.6%)	148 (31.3%)	164 (34.7%)	205 (43.3%)	
High	931 (49.2%)	248 (52.3%)	247 (52.2%)	233 (49.3%)	203 (42.9%)	
Smoking						
Non	1198 (63.3%)	315 (66.5%)	321 (67.9%)	283 (59.8%)	279 (59.0%)	0.005
Smoker	400 (21.1%)	80 (16.9%)	84 (17.8%)	117 (24.7%)	119 (25.2%)	
Ex-smoker	295 (15.6%)	79 (16.7%)	68 (14.4%)	73 (15.4%)	75 (15.9%)	
Sleep duration (hours)						
<5	274 (14.5%)	79 (16.7%)	62 (13.1%)	61 (12.9%)	72 (15.2%)	0.264
5–7	977 (51.6%)	237 (50.0%)	259 (54.8%)	241 (51.0%)	240 (50.7%)	
7–8	460 (24.3%)	121 (25.5%)	108 (22.8%)	126 (26.6%)	105 (22.2%)	
≥8	182 (9.6%)	37 (7.8%)	44 (9.3%)	45 (9.5%)	56 (11.8%)	
Snoring						
No	1266 (66.9%)	320 (67.5%)	304 (64.3%)	324 (68.5%)	318 (67.2%)	0.547
Yes	627 (33.1%)	154 (32.5%)	169 (35.7%)	149 (31.5%)	155 (32.8%)	
Leisure time physical activity (MET hours/week)	6.0 (0.0–25.5)	9.0 (0.0–30.0)	6.0 (0.0–22.5)	6.0 (0.0–27.0)	3.0 (0.0–22.8)	0.093
**Dietary intakes**						
Fruit intake (times/week)	6.0 (0.0–25.5)	9.0 (0.0–30.0)	6.0 (0.0–22.5)	6.0 (0.0–27.0)	3.0 (0.0–22.8)	0.093
Vegetable intake (times/week)	15.5 (6.5–26.5)	15.5 (6.0–25.0)	11.5 (5.5–21.5)	14.0 (7.0–24.0)	21.0 (10.0–37.0)	<0.001
Soft drink	1.0 (0.0–5.0)	0.5 (0.0–2.0)	0.5 (0.0–3.0)	2.0 (0.0–6.0)	4.0 (0.5–11.0)	<0.001
On diet						
No	468 (24.7%)	173 (36.5%)	113 (23.9%)	93 (19.7%)	89 (18.8%)	<0.001
Yes	1425 (75.3%)	301 (63.5%)	360 (76.1%)	380 (80.3%)	384 (81.2%)	
**Health outcomes**						
BMI (kg/m^2^)	31.6 (6.5)	31.8 (6.8)	32.5 (6.7)	31.5 (6.2)	30.6 (6.3)	<0.001
BMI categories						
Normal	252 (13.3%)	64 (13.5%)	49 (10.4%)	57 (12.1%)	82 (17.3%)	0.003
Overweight	601 (31.7%)	138 (29.1%)	140 (29.6%)	159 (33.6%)	164 (34.7%)	
Obese	1040 (54.9%)	272 (57.4%)	284 (60.0%)	257 (54.3%)	227 (48.0%)	
HbA1C (mmol/L)	5.5 (0.9)	5.6 (1.0)	5.6 (1.0)	5.5 (0.9)	5.4 (0.8)	0.007
HbA1C ≥ 7 (mmol/L)						
No	1762 (93.9%)	433 (91.9%)	434 (92.9%)	440 (94.0%)	455 (96.6%)	0.019
Yes	115 (6.1%)	38 (8.1%)	33 (7.1%)	28 (6.0%)	16 (3.4%)	
Hypertension						
No	1651 (87.2%)	405 (85.4%)	390 (82.5%)	426 (90.1%)	430 (90.9%)	<0.001
Yes	242 (12.8%)	69 (14.6%)	83 (17.5%)	47 (9.9%)	43 (9.1%)	
Self-reported diabetes						
No	1543 (81.5%)	371 (78.3%)	371 (78.4%)	385 (81.4%)	416 (87.9%)	<0.001
Yes	350 (18.5%)	103 (21.7%)	102 (21.6%)	88 (18.6%)	57 (12.1%)	
Diabetes						
No	1515 (80.6%)	363 (76.9%)	360 (77.1%)	378 (80.6%)	414 (87.9%)	<0.001
Yes	364 (19.4%)	109 (23.1%)	107 (22.9%)	91 (19.4%)	57 (12.1%)	
Depression symptoms						
No	1416 (74.8%)	366 (77.2%)	380 (80.3%)	350 (74.0%)	320 (67.7%)	<0.001
Yes	477 (25.2%)	108 (22.8%)	93 (19.7%)	123 (26.0%)	153 (32.3%)	
Diabetes medication other than insulin						
No	1721 (90.9%)	422 (89.0%)	415 (87.7%)	433 (91.5%)	451 (95.3%)	<0.001
Yes	172 (9.1%)	52 (11.0%)	58 (12.3%)	40 (8.5%)	22 (4.7%)	
Insulin use						
No	1817 (96.0%)	450 (94.9%)	451 (95.3%)	453 (95.8%)	463 (97.9%)	0.096
Yes	76 (4.0%)	24 (5.1%)	22 (4.7%)	20 (4.2%)	10 (2.1%)	
Hypertension medication use						
No	1714 (90.5%)	427 (90.1%)	411 (86.9%)	433 (91.5%)	443 (93.7%)	0.004
Yes	179 (9.5%)	47 (9.9%)	62 (13.1%)	40 (8.5%)	30 (6.3%)	
Weight loss (kg) *	27.6 (18.0)	27.0 (17.3)	26.2 (18.0)	28.5 (17.8)	28.8 (18.8)	0.103
Percentage of weight loss	23.4 (13.1)	23.1 (13.1)	22.0 (13.3)	23.9 (12.6)	24.8 (13.4)	0.015
Location of bariatric surgery						
Qatar	1077 (57.7%)	279 (59.7%)	270 (57.6%)	268 (57.6%)	260 (55.9%)	0.703
Outside Qatar	789 (42.3%)	188 (40.3%)	199 (42.4%)	197 (42.4%)	205 (44.1%)	

Values are mean (SD), median (IQR), or *n* (%). The *p* values were based on the χ^2^ test for categorized variable and ANOVA or Kruskal–Wallis for continuous variables among quartiles of the traditional dietary pattern (Q1, Q2, Q3 and Q4). * A total of 176 participants had missing values on weight loss.

**Table 2:** Association between dietary patterns and diabetes among adults with a history of bariatric surgery who attended the Qatar Biobank study (*n* = 1893).

	Quartiles of Dietary Pattern				
	Q1	Q2	Q3	Q4	For Trend
Traditional pattern					
Model 1	1.00	1.01 (0.73–1.41)	0.95 (0.68–1.35)	**0.65 (0.45–0.95)**	0.040
Model 2	1.00	0.95 (0.68–1.35)	0.97 (0.68–1.38)	**0.61 (0.41–0.90)**	0.029
Model 3	1.00	1.01 (0.70–1.45)	0.95 (0.65–1.39)	**0.65 (0.43–0.99)**	0.058
Prudent pattern					
Model 1	1.00	0.78 (0.53–1.13)	1.01 (0.70–1.46)	0.93 (0.64–1.35)	0.863
Model 2	1.00	0.77 (0.53–1.14)	1.00 (0.69–1.46)	0.84 (0.57–1.23)	0.698
Model 3	1.00	0.72 (0.48–1.08)	0.95 (0.64–1.41)	0.73 (0.49–1.11)	0.359
Sweet/fast food pattern					
Model 1	1.00	0.81 (0.58–1.14)	0.99 (0.70–1.39)	0.82 (0.57–1.19)	0.518
Model 2	1.00	0.89 (0.63–1.27)	1.06 (0.74–1.52)	0.90 (0.61–1.32)	0.838
Model 3	1.00	0.80 (0.54–1.16)	0.97 (0.67–1.43)	0.87 (0.58–1.30)	0.734

Values are odds ratio (95%CI) from logistic regression. Mode 1 adjusted for age and sex. Model 2 further adjusted for education, smoking, physical activity and hypertension, sleep duration and snoring. Model 3 further adjusted for quartiles of weight loss. Bold fonts represent statistically significant.

**Table 3:** Association between quartiles of dietary patterns and poor glycemic control (HbA1c ≥ 7) among adults with a history of bariatric surgery who attended the Qatar Biobank study (*n* = 1893).

	Quartiles of Dietary Pattern				
	Q1	Q2	Q3	Q4	For Trend
Traditional pattern					
Model 1	1.00	0.76 (0.45–1.28)	0.73 (0.42–1.27)	0.57 (0.30–1.09)	0.087
Model 2	1.00	0.67 (0.39–1.15)	0.76 (0.43–1.32)	**0.52 (0.27–1.00)**	0.074
Model 3	1.00	0.72 (0.41–1.27)	0.62 (0.33–1.16)	0.60 (0.30–1.20)	0.099
Prudent pattern					
Model 1	1.00	1.69 (0.83–3.43)	1.75 (0.87–3.51)	1.37 (0.67–2.81)	0.658
Model 2	1.00	1.73 (0.85–3.55)	1.81 (0.89–3.70)	1.29 (0.62–2.68)	0.846
Model 3	1.00	1.91 (0.88–4.15)	1.79 (0.82–3.91)	1.36 (0.61–3.01)	0.868
Sweet/fast food pattern					
Model 1	1.00	0.61 (0.36–1.03)	0.68 (0.40–1.17)	0.53 (0.28–0.99)	0.051
Model 2	1.00	0.67 (0.39–1.15)	0.72 (0.41–1.25)	0.57 (0.30–1.10)	0.096
Model 3	1.00	0.75 (0.41–1.37)	0.88 (0.48–1.58)	0.72 (0.36–1.45)	0.442

Values are odds ratio (95%CI) from logistic regression. Model 1 adjusted for age and sex. Model 2 further adjusted for education, smoking, physical activity and hypertension, sleep duration and snoring. Model 3 further adjusted for quartiles of weight loss. Bold fonts represent statistically significant.

**Table 4:** Subgroup analyses of the association between traditional dietary pattern and high HbA1c (HbA1c ≥ 7) among adults with a history of bariatric surgery who attended the Qatar Biobank study (*n* = 1893).

	Quartiles of Traditional					
	Q1	Q2	Q3	Q4	*p* for Trend	*p* for Interaction
Gender						0.841
Male	1.00	1.05 (0.45–2.45)	1.21 (0.52–2.80)	0.70 (0.26–1.90)	0.661	
Female	1.00	0.62 (0.31–1.23)	0.70 (0.33–1.48)	0.51 (0.21–1.20)	0.127	
Age ≥ 40 (years)						0.230
No	1.00	4.06 (0.76–21.68)	3.03 (0.56–16.34)	3.08 (0.58–16.23)	0.347	
Yes	1.00	0.62 (0.35–1.10)	0.78 (0.43–1.42)	0.38 (0.17–0.86)	0.037	
Smoking						0.378
Non	1.00	0.72 (0.38–1.36)	0.79 (0.40–1.58)	0.60 (0.27–1.30)	0.223	
Smoker	1.00	0.41 (0.10–1.63)	0.59 (0.17–2.01)	0.25 (0.05–1.34)	0.123	
Ex-smoker	1.00	4.77 (0.75–30.44)	3.72 (0.55–25.21)	1.71 (0.19–15.21)	0.687	
Leisure time PA (MET hours/week)						0.357
T1	1.00	0.36 (0.15–0.82)	0.73 (0.34–1.58)	0.53 (0.23–1.25)	0.255	
T2	1.00	1.19 (0.47–3.03)	0.86 (0.29–2.56)	0.47 (0.12–1.90)	0.297	
T3	1.00	2.50 (0.74–8.39)	1.60 (0.47–5.48)	1.22 (0.26–5.76)	0.824	
Education						0.057
Low	1.00	0.46 (0.15–1.44)	1.08 (0.39–3.05)	0.53 (0.14–1.98)	0.617	
Medium	1.00	1.53 (0.52–4.46)	2.13 (0.74–6.14)	1.64 (0.53–5.03)	0.320	
High	1.00	0.81 (0.38–1.74)	0.38 (0.14–1.04)	0.26 (0.07–0.95)	0.011	
Depression symptoms						0.547
No	1.00	0.75 (0.42–1.34)	1.01 (0.56–1.84)	0.64 (0.31–1.32)	0.425	
Yes	1.00	0.68 (0.19–2.48)	0.47 (0.10–2.16)	0.28 (0.06–1.32)	0.092	
Hypertension						0.697
No	1.00	1.01 (0.51–2.01)	0.99 (0.50–1.94)	0.63 (0.28–1.43)	0.341	
Yes	1.00	0.58 (0.26–1.31)	0.70 (0.27–1.85)	0.47 (0.16–1.37)	0.189	
On diet						0.488
No	1.00	0.55 (0.19–1.59)	0.73 (0.20–2.66)	1.01 (0.30–3.34)	0.827	
Yes	1.00	0.81 (0.43–1.52)	0.95 (0.50–1.79)	0.49 (0.22–1.07)	0.148	
Quartiles of weight loss						.
Q1	1.00	0.80 (0.33–1.94)	0.51 (0.17–1.47)	0.32 (0.09–1.10)	0.044	
Q2	1.00	0.32 (0.09–1.09)	1.05 (0.39–2.86)	0.82 (0.26–2.59)	0.937	
Q3	1.00	1.34 (0.40–4.44)	-	0.25 (0.03–2.22)	0.047	
Q4	1.00	1.12 (0.07–17.97)	3.35 (0.28–40.68)	3.91 (0.32–48.33)	0.166	
Location of bariatric surgery						0.263
Qatar	1.00	0.95 (0.45–1.99)	1.42 (0.68–2.97)	0.98 (0.42–2.31)	0.691	
Outside Qatar	1.00	0.67 (0.31–1.46)	0.53 (0.22–1.25)	0.32 (0.12–0.87)	0.019	

Values are odds ratio (95%CI) from logistic regression model. The *p* values for trend were tested using quartiles of dietary patterns as ordinal variables in the models. Models are adjusted for age, sex, education, smoking, physical activity and hypertension, sleep duration and snoring. Stratification variables were not adjusted for in corresponding models.

## Data Availability

The data that support the findings of this study are available from the Qatar Biobank study, but restrictions apply to the availability of these data, which were used under license for the current study, and are not publicly available. Data are, however, available from the authors upon reasonable request and with the permission of the Qatar Biobank study.

## Supplementary Materials

The following supporting information can be downloaded at: https://www.mdpi.com/article/10.3390/nu16142194/s1, Figure S1: Distribution of weight loss after bariatric surgery among participants attending the Qatar Biobank study (*n* = 1717); Table S1: Factor loadings of dietary patterns among adults with a history of bariatric surgery who attended the Qatar Biobank study (*n* = 1893); Table S2: Sample characteristics by diabetes remission among adults with a history of bariatric surgery and diabetes who attended the Qatar Biobank study (*n* = 350); Table S3: Association between sample characteristics and diabetes remission among Qatari adults with a history of bariatric surgery and diabetes who attended the Qatar Biobank study (*n* = 350); Table S4: Determinants of diabetes medication use and glycemic control among individuals with known diabetes and a history of bariatric surgery who attended the Qatar Biobank study (*n* = 311).
