# Supplementary material for: Association between Dietary Pattern, Weight Loss, and Diabetes among Adults with a History of Bariatric Surgery: Results from the Qatar Biobank Study
Source: Nutrients. 2024 Jul 10;16(14):2194. doi: 10.3390/nu16142194 (PMC11279436; doi:10.3390/nu16142194)
Supplement: Supplementary file 1 [file nutrients-16-02194-s001.zip › nutrients-3077637-supplementary.pdf]

Supplemental Table S1 Factor loadings of dietary patterns among adults with history of bariatric surgery who attended Qatar Biobank Study (*n*=1893)

Food	Traditional pattern	Prudent pattern	Sweet/fast food pattern
Biryani (البرياني)	0.70	0.24	0.06
Chicken/meat fish mixed dish (دجاج أو لحمة أو سمك مع الأرز)	0.63	0.34	0.20
Zaatar fatayer (فطائر بالزعتر)	0.62	0.04	0.28
Croissant	0.61	-0.02	0.33
Lasagna	0.61	0.14	0.23
Arabic/Iranian bread (خبز عربي)	0.59	0.14	0.17
White rice	0.59	0.23	-0.09
White bread	0.58	0.04	0.21
Other bread	0.51	0.04	0.25
Soups/starters	0.50	0.48	0.31
Chicken	0.48	0.15	0.00
Asian noodle	0.48	0.29	0.20
Potato	0.47	0.31	0.10
Red meat	0.44	0.20	0.07
Eggs	0.27	0.26	0.07
Tea	0.26	0.07	0.15
Fresh fruit	0.08	0.71	0.17
Salad and raw vegetables	0.22	0.69	0.10
Canned/dried fruit and dates	0.03	0.63	0.16
Grilled/fried/baked Fish	0.16	0.59	0.07
Fresh fruit juice	0.18	0.55	0.24
Fish	0.28	0.54	-0.01
Salad and cooked vegetables	0.38	0.54	0.12
Brown bread	0.32	0.33	0.13
Desserts	0.23	0.06	0.66
Ice-cream	0.15	0.09	0.65
Chocolate	0.13	-0.14	0.64
Fast food	0.51	0.14	0.54
Nuts	0.00	0.31	0.52
Cheese	0.22	0.06	0.43
Soft drink	0.35	-0.02	0.43
Milk added to cereal	-0.03	0.20	0.41
Milk shakes	0.06	0.20	0.38
Butter	0.18	0.08	0.37
Yoghurt	0.08	0.30	0.34
Coffee	0.18	0.07	0.29
Milk	0.03	0.10	0.29
Breakfast Cereal	0.16	0.17	0.23
Variance explained (%)	24.2	5.9	4.9

Supplement Table S2 Sample characteristics by diabetes remission among adults with history of bariatric surgery and diabetes who attended Qatar Biobank Study (*n* =350)

	Diabetes remission			
	No	Yes	Total	p-value
N	233 (66.6%)	117 (33.4%)	350 (100.0%)	
Age	48.7 (10.0)	40.7 (10.2)	46.1 (10.7)	<0.001
Gender				
Male	90 (38.6%)	40 (34.2%)	130 (37.1%)	0.418
Female	143 (61.4%)	77 (65.8%)	220 (62.9%)	
Education				
Low	73 (31.3%)	25 (21.4%)	98 (28.0%)	0.041
Medium	79 (33.9%)	36 (30.8%)	115 (32.9%)	
High	81 (34.8%)	56 (47.9%)	137 (39.1%)	
Smoking				
Non	162 (69.5%)	74 (63.2%)	236 (67.4%)	0.474
Smoker	40 (17.2%)	23 (19.7%)	63 (18.0%)	
Ex-smoker	31 (13.3%)	20 (17.1%)	51 (14.6%)	
Sleep duration				
<5 hours	32 (13.7%)	24 (20.5%)	56 (16.0%)	0.207
5-7 hours	117 (50.2%)	56 (47.9%)	173 (49.4%)	
7-8 hours	58 (24.9%)	30 (25.6%)	88 (25.1%)	
≥ 8 hours	26 (11.2%)	7 (6.0%)	33 (9.4%)	
Snoring				
No	135 (57.9%)	69 (59.0%)	204 (58.3%)	0.853
Yes	98 (42.1%)	48 (41.0%)	146 (41.7%)	
Leisure time physical activity (MET hours/week)	1.5 (0.0-15.0)	10.1 (0.0-30.0)	3.6 (0.0-21.0)	0.008
Leisure time physical activity (MET hours/week)				
T1	116 (49.8%)	46 (39.3%)	162 (46.3%)	0.015

T2	62 (26.6%)	26 (22.2%)	88 (25.1%)	
T3	55 (23.6%)	45 (38.5%)	100 (28.6%)	
BMI (kg/m2)	33.3 (7.1)	31.8 (5.7)	32.8 (6.7)	0.051
BMI categories				
Normal	20 (8.6%)	11 (9.4%)	31 (8.9%)	0.405
Overweight	59 (25.3%)	37 (31.6%)	96 (27.4%)	
Obese	154 (66.1%)	69 (59.0%)	223 (63.7%)	
Fruit intake (times/week)	7.5 (3.0-12.0)	6.0 (1.5-14.0)	7.0 (3.0-14.0)	0.72
Vegetable intake (times/week)	18.0 (7.5-26.5)	15.5 (6.0-27.5)	16.5 (7.0-27.0)	0.46
Soft drink	0.5 (0.0-2.0)	1.0 (0.0-7.0)	0.5 (0.0-3.0)	0.014
On diet				
No	56 (24.0%)	32 (27.4%)	88 (25.1%)	0.500
Yes	177 (76.0%)	85 (72.6%)	262 (74.9%)	
HbA1C	7.2 (1.6)	5.5 (0.4)	6.6 (1.5)	<0.001
Hypertension				
No	135 (57.9%)	94 (80.3%)	229 (65.4%)	<0.001
Yes	98 (42.1%)	23 (19.7%)	121 (34.6%)	
Depression symptom				
No	190 (81.5%)	85 (72.6%)	275 (78.6%)	0.056
Yes	43 (18.5%)	32 (27.4%)	75 (21.4%)	
Diabetes duration (years)	14.9 (9.1)	8.1 (6.4)	12.7 (8.9)	<0.001
Diabetes medication other than insulin				
No	61 (26.2%)	117 (100.0%)	178 (50.9%)	<0.001
Yes	172 (73.8%)	0 (0.0%)	172 (49.1%)	
Insulin use				
No	157 (67.4%)	117 (100.0%)	274 (78.3%)	<0.001
Yes	76 (32.6%)	0 (0.0%)	76 (21.7%)	
Hypertension medication use				
No	152 (65.2%)	100 (85.5%)	252 (72.0%)	<0.001
Yes	81 (34.8%)	17 (14.5%)	98 (28.0%)	
Weight loss (kg)	21.9 (14.2)	29.9 (19.0)	24.6 (16.3)	<0.001
Percentage of weight loss	19.2 (10.9)	24.7 (11.4)	21.0 (11.3)	<0.001

Location of bariatric surgery				
Qatar	131 (57.0%)	66 (56.9%)	197 (56.9%)	0.992
Outside Qatar	99 (43.0%)	50 (43.1%)	149 (43.1%)	
Traditional dietary pattern	-0.2 (0.8)	-0.1 (1.1)	-0.2 (0.9)	0.189
Prudent dietary pattern	0.2 (0.8)	0.1 (1.2)	0.1 (1.0)	0.548
Sweets/fast food dietary pattern	-0.2 (1.0)	0.1 (1.2)	-0.1 (1.1)	0.016

Values were mean (SD), median (IQR) or n (%).

Supplement Table S3 Association between sample characteristics and diabetes remission among Qatari adults with history of bariatric surgery and diabetes who attended Qatar Biobank Study (*n* =350)

	Model 1		Model 2		Model 3	
	OR [95% CI]	p-value	OR [95% CI]	p-value	OR [95% CI]	p-value
Age (years)	0.92 (0.90-0.95)	<0.001	0.93 (0.91-0.96)	<0.001	0.97 (0.93-1.01)	0.136
Gender						
Male	1.00		1.00		1.00	
Female	1.47 (0.88-2.43)	0.138	2.35 (1.15-4.80)	0.019	1.25 (0.52-2.98)	0.616
BMI categories						
Normal			1.00		1.00	
Overweight			2.55 (0.85-7.62)	0.094	3.17 (0.85-11.78)	0.085
Obese			1.92 (0.67-5.52)	0.227	2.24 (0.65-7.80)	0.203
Leisure time physical activity (MET hours/week)						
T1			1.00		1.00	
T2			1.22 (0.61-2.46)	0.569	1.24 (0.53-2.87)	0.617
T3			2.18 (1.13-4.19)	0.019	2.31 (1.01-5.29)	0.048
Smoking						
Non			1.00		1.00	
Smoker			0.83 (0.35-1.93)	0.658	0.40 (0.14-1.18)	0.097
Ex-smoker			1.82 (0.79-4.17)	0.158	1.40 (0.51-3.82)	0.510
Quartiles of weight loss						
Q1 (low)			1.00		1.00	
Q2			1.57 (0.76-3.25)	0.220	2.56 (0.98-6.70)	0.055
Q3			2.88 (1.30-6.35)	0.009	5.38 (1.98-14.64)	<0.001
Q4 (high)			3.96 (1.61-9.72)	0.003	5.94 (1.89-18.69)	0.002
Traditional dietary pattern			1.10 (0.79-1.54)	0.573	0.99 (0.66-1.50)	0.966
Prudent dietary pattern			1.09 (0.82-1.46)	0.538	1.24 (0.85-1.82)	0.259
Sweets/fast food pattern			1.25 (0.97-1.61)	0.084	1.55 (1.11-2.17)	0.010
Diabetes duration (years)					0.90 (0.85-0.95)	<0.001
N	350		311		237	

Variables in the models were mutually adjusted.

Supplemental Table S4 Determinants of diabetes medication use, glycemic control among individuals with known diabetes and history of bariatry surgery who attended Qatar Biobank Study (*n* =311)

	Insulin use		Other diabetes medication use		HbA1c ≥7%	
	OR [95% CI]	p-value	OR. [95% CI]	p-value	OR. [95% CI]	p-value
Traditional pattern	0.86 (0.59-1.26)	0.440	0.81 (0.59-1.12)	0.202	0.86 (0.62-1.19)	0.366
Prudent pattern	0.98 (0.70-1.36)	0.898	0.96 (0.72-1.29)	0.806	1.03 (0.77-1.39)	0.845
Sweets/fast food pattern	1.16 (0.88-1.53)	0.302	**0.63 (0.46-0.85)**	**0.003**	0.84 (0.63-1.12)	0.224
Quartiles of weight loss						
Q1	1.00		1.00		1.00	
Q2	0.51 (0.24-1.07)	0.076	1.17 (0.60-2.29)	0.640	0.77 (0.41-1.47)	0.435
Q3	0.34 (0.15-0.81)	0.015	**0.47 (0.23-0.97)**	**0.040**	**0.28 (0.13-0.61)**	**0.001**
Q4	0.47 (0.18-1.25)	0.131	0.72 (0.31-1.67)	0.448	**0.28 (0.11-0.69)**	**0.006**
Age	1.02 (0.98-1.06)	0.334	1.07 (1.03-1.10)	<0.001	1.04 (1.00-1.07)	0.038
Gender						
Male	1.00		1.00		1.00	
Female	0.42 (0.19-0.89)	0.024	1.42 (0.73-2.74)	0.302	0.39 (0.20-0.78)	0.007
Education						
Low	1.00		1.00		1.00	
Medium	1.99 (0.83-4.76)	0.121	2.64 (1.18-5.90)	0.018	2.04 (0.92-4.55)	0.080
High	0.79 (0.33-1.87)	0.594	1.47 (0.71-3.03)	0.302	1.62 (0.77-3.43)	0.206
Smoking						
Non	1.00		1.00		1.00	
Smoker	0.42 (0.15-1.15)	0.091	1.14 (0.52-2.53)	0.741	1.08 (0.47-2.46)	0.864
Ex-smoker	0.66 (0.26-1.63)	0.366	0.58 (0.26-1.30)	0.188	0.77 (0.34-1.75)	0.536
Hypertension						
No	1.00		1.00		1.00	
Yes	1.47 (0.78-2.77)	0.236	1.45 (0.83-2.53)	0.188	0.99 (0.56-1.75)	0.973
Leisure time physical activity (MET hours/week)						
T1	1.00		1.00		1.00	

T2	0.72 (0.33-1.56)	0.401	1.18 (0.61-2.27)	0.627	0.96 (0.48-1.89)	0.899
T3	0.69 (0.32-1.49)	0.349	0.54 (0.28-1.04)	0.065	0.59 (0.30-1.17)	0.133

Variables in the table were mutually adjusted.

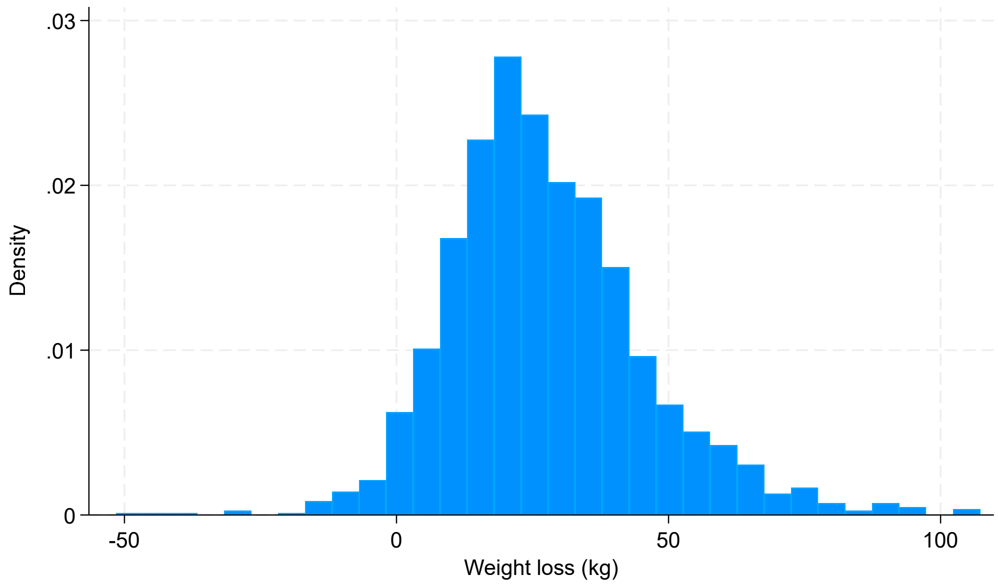

Supplement Figure S1 Distribution of weight loss after bariatric surgery among participants attending Qatar Biobank Study (*n* =1717)
